# Methyl (2*Z*)-((2*Z*)-2-{(2*E*)-[1-(4-methyl­phen­yl)ethyl­idene]hydrazinyl­idene}-4-oxo-3-phenyl-1,3-thia­zolidin-5-yl­idene)ethano­ate

**DOI:** 10.1107/S1600536813021533

**Published:** 2013-08-10

**Authors:** Joel T. Mague, Mehmet Akkurt, Shaaban K. Mohamed, Alaa A. Hassan, Mustafa R. Albayati

**Affiliations:** a Department of Chemistry, Tulane University, New Orleans, LA 70118, USA; b Department of Physics, Faculty of Sciences, Erciyes University, 38039 Kayseri, Turkey; c Chemistry and Environmental Division, Manchester Metropolitan University, Manchester, M1 5GD, England; d Chemistry Department, Faculty of Science, Minia University, 61519 El-Minia, Egypt; e Kirkuk University, College of Science, Department of Chemistry, Kirkuk, Iraq

## Abstract

The asymmetric unit of the title compound, C_21_H_19_N_3_O_3_S, contains two independent mol­ecules. In one mol­ecule, the 1,3-thia­zolidine ring forms dihedral angles of 86.19 (8) and 8.37 (8)° with the phenyl and benzene rings, respectively. The corresponding dihedral angles in the other mol­ecule are 69.60 (7) and 14.08 (7)°. The dihedral angle between the phenyl and benzene rings is 84.70 (8)° in one mol­ecule and 69.62 (8)° in the other. In the crystal, mol­ecules pack in layers approximately parallel to (10-2). There are weak C—H⋯O hydrogen bonds within these layers. Further weak C—H⋯O hydrogen bonding occurs between the layers to form a three-dimensional network. A weak C—H⋯π inter­action is also observed.

## Related literature

For the synthesis and general applications of thia­zolidines, see: Pandey *et al.* (2011 ▶); Barreca *et al.* (2002 ▶); Botti *et al.* (1996 ▶); Pfahl *et al.* (2003 ▶); Sayyed *et al.* (2006 ▶); Sharma *et al.* (2006 ▶); Babaoğlu *et al.* (2003 ▶); Çapan *et al.* (1999 ▶). For standard bond-length data, see: Allen *et al.* (1987 ▶).
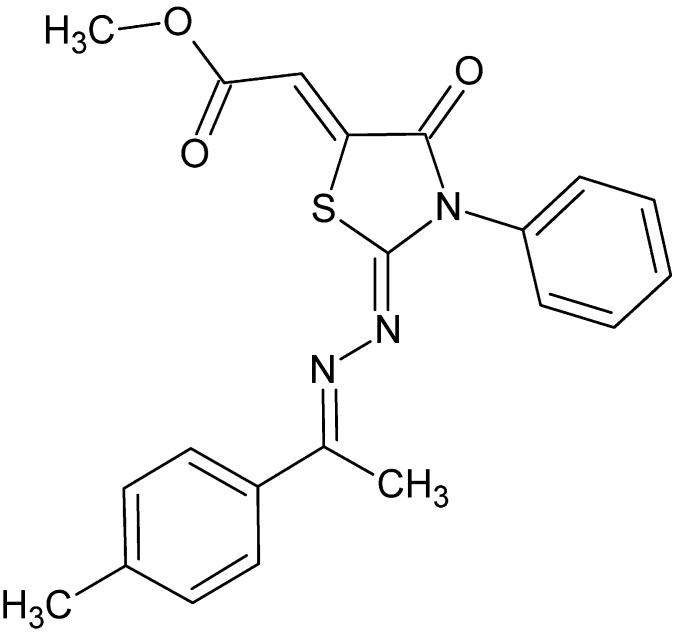


## Experimental

### 

#### Crystal data

C_21_H_19_N_3_O_3_S
*M* *_r_* = 393.46
Monoclinic, 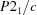

*a* = 8.3713 (12) Å
*b* = 21.568 (3) Å
*c* = 21.591 (3) Å
β = 96.411 (2)°
*V* = 3873.9 (9) Å^3^
*Z* = 8
Mo *K*α radiation
μ = 0.19 mm^−1^
*T* = 150 K
0.19 × 0.19 × 0.11 mm

#### Data collection

Bruker SMART APEX CCD diffractometer
Absorption correction: multi-scan (*SADABS*; Bruker, 2013 ▶) *T*_min_ = 0.81, *T*_max_ = 0.98
69242 measured reflections
9850 independent reflections
7890 reflections with *I* > 2σ(*I*)
*R*_int_ = 0.057

#### Refinement

*R*[*F* ^2^ > 2σ(*F* ^2^)] = 0.044
*wR*(*F* ^2^) = 0.112
*S* = 1.05
9850 reflections
511 parameters
H-atom parameters constrained
Δρ_max_ = 0.34 e Å^−3^
Δρ_min_ = −0.34 e Å^−3^

### 

Data collection: *APEX2* (Bruker, 2013 ▶); cell refinement: *SAINT* (Bruker, 2013 ▶); data reduction: *SAINT*; program(s) used to solve structure: *SHELXS97* (Sheldrick, 2008 ▶); program(s) used to refine structure: *SHELXL97* (Sheldrick, 2008 ▶); molecular graphics: *ORTEP-3 for Windows* (Farrugia, 2012 ▶) and *DIAMOND* (Brandenburg & Putz, 2012 ▶).; software used to prepare material for publication: *WinGX* (Farrugia, 2012 ▶) and *PLATON* (Spek, 2009 ▶).

## Supplementary Material

Crystal structure: contains datablock(s) global, I. DOI: 10.1107/S1600536813021533/lh5636sup1.cif

Structure factors: contains datablock(s) I. DOI: 10.1107/S1600536813021533/lh5636Isup2.hkl

Supplementary material file. DOI: 10.1107/S1600536813021533/lh5636Isup3.cml

Additional supplementary materials:  crystallographic information; 3D view; checkCIF report

## Figures and Tables

**Table 1:** Hydrogen-bond geometry (Å, °) *Cg* is the centroid of the C36–C41 ring.

*D*—H⋯*A*	*D*—H	H⋯*A*	*D*⋯*A*	*D*—H⋯*A*
C4—H4⋯O6^i^	0.95	2.56	3.4802 (19)	163
C6—H6*B*⋯O4^i^	0.98	2.52	3.465 (2)	163
C8—H8⋯O2^ii^	0.95	2.56	3.359 (2)	142
C12—H12⋯O4	0.95	2.43	3.302 (2)	152
C27—H27*B*⋯O1^i^	0.98	2.45	3.410 (2)	166
C30—H30⋯O5^iii^	0.95	2.44	3.331 (2)	157
C32—H32⋯O1^iv^	0.95	2.57	3.340 (2)	139
C33—H33⋯*Cg* ^v^	0.95	2.58	3.4951 (19)	162

Symmetry codes: (i) 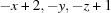; (ii) 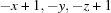; (iii) 
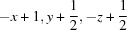
; (iv) 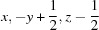; (v) 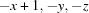.

**Fig. 1.**
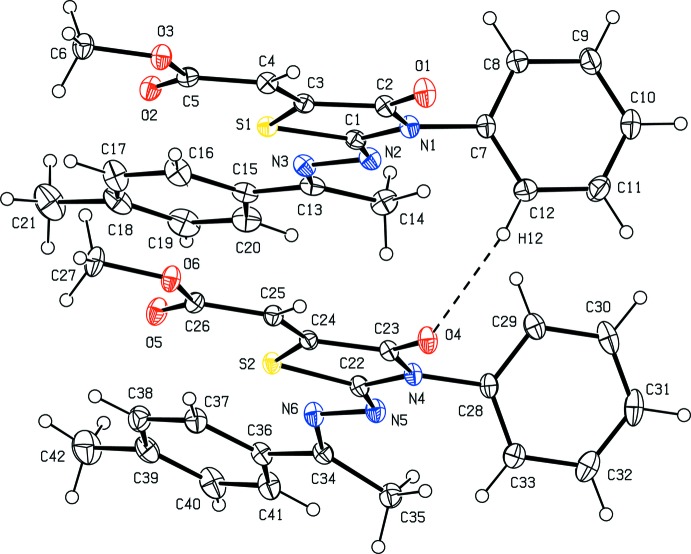
The asymmetric unit of (I) showing a weak C—H···O hydrogen bond (dashed line) between the independent molecules. Displacement ellipsoids are at the 30% probabilty level.

**Fig. 2.**
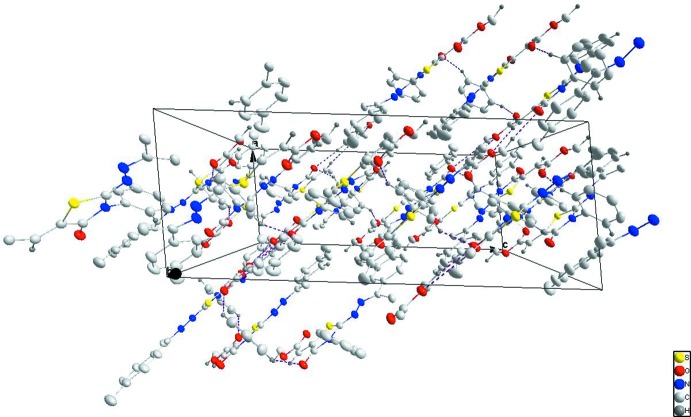
Part of the crystal structure viewed approximately along the b axis with weak C—H···O hydrogen bonds shown as dashed lines.

**Fig. 3.**
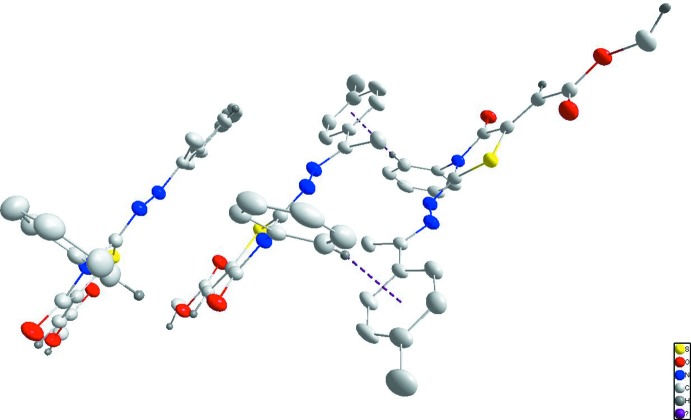
A view along the b axis showing weak C—H···π interactions as dashed lines.

## supplementary crystallographic information

### 1. Comment

Heterocyclic compounds such as thiazolidines have demonstrated widespread applications as pharmaceuticals (Pandey *et al.*, 2011; Barreca *et al.*, 2002) and materials (Botti *et al.*, 1996). Thiazolidinone derivatives exhibit anticancer properties (Pfahl *et al*., 2003) as well as antibacterial (Sayyed *et al.*, 2006), antimycobacterial (Babaoğlu *et al.*, 2003), antimicrobial (Sharma *et al.*, 2006) and antifungal (Çapan *et al.*, 1999) activities. Therefore, we have synthesized the title compound (I) and determined its crystal structure.

The asymmetric unit of (I) is shown in Fig. 1. In one molecule, the 1,3-thiazolidine ring (S1/N1/C1–C3) forms dihedral angles of 86.19 (8) and 8.37 (8) °, respectively, with the phenyl ring (C7–C12) and the benzene ring (C15–C20). In the other molecule the corresponding angles between (S2/N4/C22-C24) are 69.60 (7) (C28-C33) and 14.08 (7)° (C36-C41), respectively. The dihedral angle between the phenyl and benzene rings is 84.70 (8)° (C7-C12 and C15-C20) and 69.62 (8)° (C28-C33 and C26-C41). The differences in the dihedral angles may be due to the packing effects of neighbouring molecules. The bond lengths (Allen *et al.*, 1987) and angles in each molecule are normal.

In the crystal, molecules are arranged in layers approximately parallel to (102) connected by weak C—H···O hydrogen bonds (Table 1, Fig. 2). Additional weak C—H···O hydrogen bonds occur between theses layers (Table 1, Fig. 2) forming a three-dimensional network. A weak intermolecular C—H···π interaction is also observed (Table 1).

### 2. Experimental

A mixture of 283 mg (1 mmol) (2E)-2-[1-(4-methylphenyl)ethylidene]-*N*-phenylhydrazinecarbothioamide and 142 mg (1 mmol) of dimethyl but-2-ynedioate in 50 ml methanol was refluxed for three hours. The excess solvent was evaporated under vacuum and the residual solid product was collected and recrystallized from ethanol to afford a clear yellow blocks (*M*.p. 527 – 529 K) suitable for X-ray diffraction.

### 3. Refinement

H atoms were placed in geometrically idealized positions and constrained to ride on their parent atoms with C—H = 0.95 (aromatic H) and 0.98 Å (methyl H), with *U*_iso_(H) = 1.2 *U*_iso_(C) for aromatic H atoms and *U*_iso_(H) = 1.5 *U*_iso_(C) for methyl H atoms.

### Crystal data

**Table:**

C_21_H_19_N_3_O_3_S	*F*(000) = 1648
*M**_r_* = 393.46	*D*_x_ = 1.349 Mg m^−^^3^
Monoclinic, *P*2_1_/*c*	Mo *K*α radiation, λ = 0.71073 Å
Hall symbol: -P 2ybc	Cell parameters from 9179 reflections
*a* = 8.3713 (12) Å	θ = 2.5–28.6°
*b* = 21.568 (3) Å	µ = 0.19 mm^−^^1^
*c* = 21.591 (3) Å	*T* = 150 K
β = 96.411 (2)°	Block, clear yellow
*V* = 3873.9 (9) Å^3^	0.19 × 0.19 × 0.11 mm
*Z* = 8	

### Data collection

**Table:**

Bruker SMART APEX CCD diffractometer	9850 independent reflections
Radiation source: fine-focus sealed tube	7890 reflections with *I* > 2σ(*I*)
Graphite monochromator	*R*_int_ = 0.057
Detector resolution: 8.3660 pixels mm^-1^	θ_max_ = 28.6°, θ_min_ = 1.9°
φ and ω scans	*h* = −10→11
Absorption correction: multi-scan (*SADABS*; Bruker, 2013)	*k* = −28→28
*T*_min_ = 0.81, *T*_max_ = 0.98	*l* = −28→28
69242 measured reflections	

### Refinement

**Table:**

Refinement on *F*^2^	0 restraints
Least-squares matrix: full	Hydrogen site location: inferred from neighbouring sites
*R*[*F*^2^ > 2σ(*F*^2^)] = 0.044	H-atom parameters constrained
*wR*(*F*^2^) = 0.112	W = 1/[Σ^2^(*F*O^2^) + (0.0511*P*)^2^ + 1.054*P*] WHERE *P* = (*F*O^2^ + 2*F*C^2^)/3
*S* = 1.05	(Δ/σ)_max_ = 0.001
9850 reflections	Δρ_max_ = 0.34 e Å^−^^3^
511 parameters	Δρ_min_ = −0.34 e Å^−^^3^

### Special details

**Table:**

Experimental. ^1^H-NMR [δ, p.p.m., CDCl_3_]: 2.27 (S, 3H, CH_3_), 2.39 (S, 3H, CH_3_), 3.88 (S, 3H, OCH_3_), 6.97 (S, 1H, vinyl - CH), 7.19–7.26 (m, 2H, Ar- H), 7.44–7.47 (m, 3H, Ar - H), 7.51–7.55 (m, 2H, Ar - H), 7.78–7.83 (m, 2H, Ar - H). ^13^C-NMR [δ, p.p.m., CDCl_3_]: 15.03 (CH_3_), 21.42 (CH_3_), 52.49 (OCH_3_), 115.98 (vinyl-CH), 126.92, 127.46, 128.90, 129.09, 129.30 (Ar - CH), 132.21, 133.76 (Ar - C), 140.75 (Ar – C– N), 142.05 (CH_3_ – C=N), 158.15 (thiazole – C.2), 164.9 (cyclic – C=O), 166.59 (ester - CO).
Geometry. Bond distances, angles *etc*. have been calculated using the rounded fractional coordinates. All su's are estimated from the variances of the (full) variance-covariance matrix. The cell e.s.d.'s are taken into account in the estimation of distances, angles and torsion angles
Refinement. Refinement on *F*^2^ for ALL reflections except those flagged by the user for potential systematic errors. Weighted *R*-factors *wR* and all goodnesses of fit *S* are based on *F*^2^, conventional *R*-factors *R* are based on *F*, with *F* set to zero for negative *F*^2^. The observed criterion of *F*^2^ > σ(*F*^2^) is used only for calculating -*R*-factor-obs *etc*. and is not relevant to the choice of reflections for refinement. *R*-factors based on *F*^2^ are statistically about twice as large as those based on *F*, and *R*-factors based on ALL data will be even larger.

### Fractional atomic coordinates and isotropic or equivalent isotropic displacement parameters (Å^2^)

**Table:**

	*x*	*y*	*z*	*U*_iso_*/*U*_eq_	
S1	0.59531 (4)	0.00119 (2)	0.42718 (2)	0.0226 (1)	
O1	0.79183 (14)	0.14802 (5)	0.50471 (5)	0.0337 (3)	
O2	0.74230 (14)	−0.08842 (5)	0.50854 (5)	0.0302 (3)	
O3	0.93261 (13)	−0.06779 (5)	0.58848 (5)	0.0285 (3)	
N1	0.60347 (15)	0.12318 (6)	0.42279 (6)	0.0241 (3)	
N2	0.42172 (15)	0.07810 (6)	0.34568 (6)	0.0263 (4)	
N3	0.36391 (15)	0.01878 (6)	0.32675 (6)	0.0260 (4)	
C1	0.52809 (17)	0.07228 (7)	0.39283 (7)	0.0228 (4)	
C2	0.71496 (18)	0.10956 (7)	0.47312 (7)	0.0250 (4)	
C3	0.72573 (17)	0.04065 (6)	0.48243 (7)	0.0227 (4)	
C4	0.82274 (18)	0.01562 (7)	0.52956 (7)	0.0247 (4)	
C5	0.82622 (18)	−0.05181 (7)	0.53990 (7)	0.0244 (4)	
C6	0.9370 (2)	−0.13330 (7)	0.60373 (8)	0.0319 (5)	
C7	0.55457 (18)	0.18631 (6)	0.40694 (7)	0.0244 (4)	
C8	0.4378 (2)	0.21376 (7)	0.43831 (8)	0.0306 (5)	
C9	0.3904 (2)	0.27400 (8)	0.42290 (9)	0.0368 (5)	
C10	0.4589 (2)	0.30597 (7)	0.37721 (8)	0.0367 (5)	
C11	0.5760 (2)	0.27798 (8)	0.34637 (8)	0.0389 (6)	
C12	0.6251 (2)	0.21778 (8)	0.36116 (8)	0.0334 (5)	
C13	0.25712 (18)	0.01879 (7)	0.27854 (7)	0.0260 (4)	
C14	0.1978 (2)	0.07602 (8)	0.24368 (8)	0.0346 (5)	
C15	0.19100 (18)	−0.04256 (8)	0.25778 (7)	0.0279 (4)	
C16	0.2319 (2)	−0.09620 (8)	0.29201 (8)	0.0367 (5)	
C17	0.1656 (2)	−0.15299 (8)	0.27352 (9)	0.0408 (6)	
C18	0.0564 (2)	−0.15848 (9)	0.22020 (9)	0.0390 (6)	
C19	0.0179 (2)	−0.10561 (9)	0.18561 (9)	0.0436 (6)	
C20	0.0840 (2)	−0.04854 (9)	0.20358 (8)	0.0373 (5)	
C21	−0.0198 (3)	−0.22033 (10)	0.20217 (11)	0.0575 (8)	
S2	0.57525 (4)	−0.01716 (2)	0.19020 (2)	0.0234 (1)	
O4	0.79280 (13)	0.12820 (5)	0.26255 (5)	0.0291 (3)	
O5	0.70484 (14)	−0.10494 (5)	0.27998 (5)	0.0326 (3)	
O6	0.89186 (13)	−0.08142 (5)	0.36026 (5)	0.0286 (3)	
N4	0.60388 (15)	0.10390 (5)	0.18031 (6)	0.0229 (3)	
N5	0.42977 (16)	0.06042 (6)	0.10082 (6)	0.0266 (4)	
N6	0.35687 (16)	0.00405 (6)	0.08093 (6)	0.0266 (4)	
C22	0.52529 (17)	0.05328 (6)	0.15065 (7)	0.0230 (4)	
C23	0.70949 (17)	0.09035 (6)	0.23226 (7)	0.0222 (4)	
C24	0.70620 (17)	0.02231 (6)	0.24547 (7)	0.0221 (4)	
C25	0.79655 (17)	−0.00146 (6)	0.29468 (7)	0.0230 (4)	
C26	0.79111 (18)	−0.06797 (7)	0.30954 (7)	0.0239 (4)	
C27	0.8927 (2)	−0.14517 (7)	0.38080 (8)	0.0350 (5)	
C28	0.57567 (17)	0.16650 (6)	0.15832 (7)	0.0244 (4)	
C29	0.49625 (19)	0.20722 (7)	0.19350 (8)	0.0313 (5)	
C30	0.4714 (2)	0.26784 (8)	0.17250 (9)	0.0401 (6)	
C31	0.5262 (2)	0.28624 (8)	0.11736 (9)	0.0429 (6)	
C32	0.6065 (2)	0.24503 (8)	0.08284 (9)	0.0415 (6)	
C33	0.6307 (2)	0.18437 (7)	0.10301 (8)	0.0329 (5)	
C34	0.28762 (18)	0.00516 (7)	0.02447 (7)	0.0246 (4)	
C35	0.2913 (2)	0.05937 (7)	−0.01919 (7)	0.0321 (5)	
C36	0.19741 (18)	−0.05168 (7)	0.00294 (7)	0.0257 (4)	
C37	0.2025 (2)	−0.10475 (7)	0.03991 (8)	0.0310 (5)	
C38	0.1130 (2)	−0.15691 (8)	0.02074 (8)	0.0358 (5)	
C39	0.0141 (2)	−0.15761 (8)	−0.03558 (8)	0.0360 (5)	
C40	0.0101 (2)	−0.10492 (8)	−0.07239 (9)	0.0404 (5)	
C41	0.1002 (2)	−0.05263 (8)	−0.05402 (8)	0.0343 (5)	
C42	−0.0846 (3)	−0.21374 (10)	−0.05661 (11)	0.0567 (8)	
H4	0.89000	0.04180	0.55660	0.0300*	
H6A	0.83230	−0.14610	0.61550	0.0480*	
H6B	1.02020	−0.14080	0.63860	0.0480*	
H6C	0.96160	−0.15720	0.56740	0.0480*	
H8	0.39080	0.19180	0.46980	0.0370*	
H9	0.31010	0.29340	0.44400	0.0440*	
H10	0.42570	0.34720	0.36690	0.0440*	
H11	0.62300	0.30010	0.31490	0.0470*	
H12	0.70590	0.19850	0.34020	0.0400*	
H14A	0.24530	0.11280	0.26520	0.0520*	
H14B	0.08040	0.07820	0.24180	0.0520*	
H14C	0.22890	0.07450	0.20130	0.0520*	
H16	0.30630	−0.09370	0.32850	0.0440*	
H17	0.19520	−0.18890	0.29760	0.0490*	
H19	−0.05520	−0.10840	0.14880	0.0520*	
H20	0.05610	−0.01300	0.17870	0.0450*	
H21A	−0.12960	−0.22130	0.21390	0.0860*	
H21B	0.04350	−0.25360	0.22390	0.0860*	
H21C	−0.02270	−0.22620	0.15700	0.0860*	
H25	0.86560	0.02510	0.32060	0.0280*	
H27A	0.79620	−0.15330	0.40140	0.0530*	
H27B	0.98860	−0.15280	0.41020	0.0530*	
H27C	0.89380	−0.17270	0.34480	0.0530*	
H29	0.45920	0.19410	0.23140	0.0380*	
H30	0.41680	0.29650	0.19610	0.0480*	
H31	0.50860	0.32760	0.10310	0.0510*	
H32	0.64510	0.25830	0.04530	0.0500*	
H33	0.68420	0.15560	0.07920	0.0400*	
H35A	0.35810	0.09240	0.00130	0.0480*	
H35B	0.18180	0.07490	−0.03020	0.0480*	
H35C	0.33650	0.04620	−0.05700	0.0480*	
H37	0.26800	−0.10530	0.07880	0.0370*	
H38	0.11930	−0.19270	0.04650	0.0430*	
H40	−0.05590	−0.10450	−0.11120	0.0480*	
H41	0.09570	−0.01730	−0.08040	0.0410*	
H42A	−0.19850	−0.20230	−0.06310	0.0850*	
H42B	−0.06860	−0.24610	−0.02470	0.0850*	
H42C	−0.05080	−0.22930	−0.09580	0.0850*	

### Atomic displacement parameters (Å^2^)

**Table:**

	*U*^11^	*U*^22^	*U*^33^	*U*^12^	*U*^13^	*U*^23^
S1	0.0235 (2)	0.0238 (2)	0.0199 (2)	−0.0006 (1)	−0.0002 (1)	−0.0011 (1)
O1	0.0348 (6)	0.0278 (6)	0.0350 (6)	−0.0002 (5)	−0.0117 (5)	−0.0044 (5)
O2	0.0365 (6)	0.0277 (5)	0.0250 (5)	−0.0039 (4)	−0.0030 (5)	0.0016 (4)
O3	0.0288 (6)	0.0262 (5)	0.0286 (6)	0.0011 (4)	−0.0055 (4)	0.0035 (4)
N1	0.0253 (6)	0.0225 (6)	0.0230 (6)	0.0015 (5)	−0.0033 (5)	−0.0004 (5)
N2	0.0260 (7)	0.0280 (6)	0.0239 (6)	0.0021 (5)	−0.0021 (5)	−0.0034 (5)
N3	0.0259 (7)	0.0287 (6)	0.0224 (6)	0.0013 (5)	−0.0022 (5)	−0.0040 (5)
C1	0.0225 (7)	0.0253 (7)	0.0203 (7)	0.0018 (5)	0.0012 (5)	−0.0021 (5)
C2	0.0244 (7)	0.0257 (7)	0.0241 (7)	0.0016 (5)	−0.0005 (6)	−0.0020 (6)
C3	0.0209 (7)	0.0244 (7)	0.0229 (7)	0.0003 (5)	0.0031 (6)	−0.0014 (5)
C4	0.0227 (7)	0.0268 (7)	0.0240 (7)	−0.0003 (5)	−0.0006 (6)	−0.0012 (6)
C5	0.0217 (7)	0.0288 (7)	0.0228 (7)	−0.0004 (5)	0.0024 (6)	0.0006 (6)
C6	0.0347 (9)	0.0277 (8)	0.0318 (8)	0.0031 (6)	−0.0025 (7)	0.0054 (6)
C7	0.0268 (8)	0.0212 (7)	0.0235 (7)	0.0003 (5)	−0.0048 (6)	−0.0017 (5)
C8	0.0328 (9)	0.0275 (7)	0.0320 (8)	−0.0003 (6)	0.0056 (7)	−0.0009 (6)
C9	0.0357 (9)	0.0275 (8)	0.0477 (10)	0.0042 (6)	0.0068 (8)	−0.0027 (7)
C10	0.0419 (10)	0.0241 (7)	0.0417 (10)	0.0013 (7)	−0.0053 (8)	0.0029 (7)
C11	0.0494 (11)	0.0340 (9)	0.0335 (9)	−0.0030 (7)	0.0052 (8)	0.0083 (7)
C12	0.0386 (9)	0.0335 (8)	0.0288 (8)	0.0021 (7)	0.0063 (7)	0.0011 (6)
C13	0.0202 (7)	0.0371 (8)	0.0203 (7)	0.0019 (6)	0.0012 (6)	−0.0025 (6)
C14	0.0290 (9)	0.0438 (9)	0.0292 (8)	0.0007 (7)	−0.0052 (7)	0.0053 (7)
C15	0.0202 (7)	0.0394 (8)	0.0237 (7)	0.0007 (6)	0.0009 (6)	−0.0057 (6)
C16	0.0290 (9)	0.0410 (9)	0.0373 (9)	0.0027 (7)	−0.0090 (7)	−0.0070 (7)
C17	0.0321 (9)	0.0367 (9)	0.0505 (11)	0.0038 (7)	−0.0087 (8)	−0.0081 (8)
C18	0.0266 (9)	0.0456 (10)	0.0437 (10)	0.0008 (7)	−0.0006 (7)	−0.0190 (8)
C19	0.0362 (10)	0.0593 (12)	0.0323 (9)	−0.0035 (8)	−0.0091 (8)	−0.0150 (8)
C20	0.0338 (9)	0.0501 (10)	0.0260 (8)	−0.0007 (7)	−0.0061 (7)	−0.0025 (7)
C21	0.0447 (12)	0.0523 (12)	0.0720 (15)	−0.0036 (9)	−0.0093 (11)	−0.0260 (11)
S2	0.0273 (2)	0.0195 (2)	0.0226 (2)	−0.0013 (1)	−0.0010 (1)	−0.0012 (1)
O4	0.0307 (6)	0.0212 (5)	0.0330 (6)	−0.0013 (4)	−0.0072 (5)	−0.0031 (4)
O5	0.0379 (7)	0.0259 (5)	0.0317 (6)	−0.0070 (5)	−0.0066 (5)	0.0031 (4)
O6	0.0309 (6)	0.0226 (5)	0.0301 (6)	−0.0011 (4)	−0.0060 (5)	0.0046 (4)
N4	0.0250 (6)	0.0187 (5)	0.0240 (6)	0.0015 (4)	−0.0021 (5)	−0.0007 (4)
N5	0.0292 (7)	0.0230 (6)	0.0261 (6)	0.0001 (5)	−0.0032 (5)	−0.0021 (5)
N6	0.0297 (7)	0.0224 (6)	0.0257 (6)	0.0007 (5)	−0.0054 (5)	−0.0019 (5)
C22	0.0227 (7)	0.0213 (6)	0.0244 (7)	0.0007 (5)	0.0005 (6)	−0.0026 (5)
C23	0.0218 (7)	0.0207 (6)	0.0237 (7)	0.0016 (5)	0.0012 (5)	−0.0020 (5)
C24	0.0227 (7)	0.0197 (6)	0.0242 (7)	−0.0003 (5)	0.0042 (6)	−0.0025 (5)
C25	0.0231 (7)	0.0222 (7)	0.0232 (7)	−0.0019 (5)	0.0009 (6)	−0.0016 (5)
C26	0.0244 (7)	0.0238 (7)	0.0231 (7)	0.0000 (5)	0.0016 (6)	0.0024 (5)
C27	0.0426 (10)	0.0242 (7)	0.0352 (9)	−0.0027 (6)	−0.0092 (7)	0.0092 (6)
C28	0.0225 (7)	0.0197 (6)	0.0292 (8)	0.0011 (5)	−0.0048 (6)	−0.0001 (5)
C29	0.0295 (8)	0.0274 (7)	0.0354 (9)	0.0036 (6)	−0.0032 (7)	−0.0042 (6)
C30	0.0388 (10)	0.0264 (8)	0.0513 (11)	0.0094 (7)	−0.0116 (8)	−0.0097 (7)
C31	0.0444 (11)	0.0224 (8)	0.0560 (12)	−0.0003 (7)	−0.0201 (9)	0.0070 (7)
C32	0.0421 (10)	0.0363 (9)	0.0437 (10)	−0.0055 (7)	−0.0057 (8)	0.0135 (8)
C33	0.0345 (9)	0.0301 (8)	0.0338 (9)	0.0013 (6)	0.0021 (7)	0.0037 (6)
C34	0.0225 (7)	0.0256 (7)	0.0245 (7)	0.0048 (5)	−0.0021 (6)	−0.0016 (5)
C35	0.0388 (9)	0.0296 (8)	0.0265 (8)	0.0016 (6)	−0.0020 (7)	0.0011 (6)
C36	0.0250 (7)	0.0260 (7)	0.0245 (7)	0.0047 (5)	−0.0042 (6)	−0.0041 (6)
C37	0.0357 (9)	0.0292 (8)	0.0262 (8)	−0.0001 (6)	−0.0049 (7)	−0.0027 (6)
C38	0.0444 (10)	0.0290 (8)	0.0332 (9)	−0.0025 (7)	0.0015 (7)	−0.0020 (7)
C39	0.0358 (9)	0.0330 (8)	0.0379 (9)	−0.0004 (7)	−0.0014 (7)	−0.0134 (7)
C40	0.0418 (10)	0.0393 (9)	0.0352 (9)	0.0051 (7)	−0.0168 (8)	−0.0120 (7)
C41	0.0404 (10)	0.0305 (8)	0.0286 (8)	0.0068 (7)	−0.0105 (7)	−0.0020 (6)
C42	0.0638 (14)	0.0453 (11)	0.0589 (14)	−0.0164 (10)	−0.0026 (11)	−0.0202 (10)

### Geometric parameters (Å, º)

**Table:**

S1—C1	1.7676 (16)	C11—H11	0.9500
S1—C3	1.7460 (15)	C12—H12	0.9500
S2—C22	1.7699 (14)	C14—H14A	0.9800
S2—C24	1.7488 (15)	C14—H14B	0.9800
O1—C2	1.2122 (19)	C14—H14C	0.9800
O2—C5	1.2121 (19)	C16—H16	0.9500
O3—C5	1.3430 (19)	C17—H17	0.9500
O3—C6	1.4503 (19)	C19—H19	0.9500
O4—C23	1.2160 (18)	C20—H20	0.9500
O5—C26	1.2090 (19)	C21—H21B	0.9800
O6—C26	1.3373 (19)	C21—H21C	0.9800
O6—C27	1.4445 (19)	C21—H21A	0.9800
N1—C7	1.4517 (19)	C23—C24	1.4958 (19)
N1—C1	1.390 (2)	C24—C25	1.336 (2)
N1—C2	1.383 (2)	C25—C26	1.472 (2)
N2—C1	1.282 (2)	C28—C29	1.380 (2)
N2—N3	1.4121 (18)	C28—C33	1.382 (2)
N3—C13	1.294 (2)	C29—C30	1.392 (2)
N4—C22	1.3931 (18)	C30—C31	1.381 (3)
N4—C28	1.4419 (17)	C31—C32	1.382 (3)
N4—C23	1.380 (2)	C32—C33	1.387 (2)
N5—C22	1.276 (2)	C34—C35	1.504 (2)
N5—N6	1.4059 (19)	C34—C36	1.487 (2)
N6—C34	1.290 (2)	C36—C37	1.393 (2)
C2—C3	1.501 (2)	C36—C41	1.397 (2)
C3—C4	1.342 (2)	C37—C38	1.389 (2)
C4—C5	1.471 (2)	C38—C39	1.393 (2)
C7—C12	1.384 (2)	C39—C40	1.385 (2)
C7—C8	1.383 (2)	C39—C42	1.507 (3)
C8—C9	1.388 (2)	C40—C41	1.390 (2)
C9—C10	1.380 (2)	C25—H25	0.9500
C10—C11	1.384 (2)	C27—H27A	0.9800
C11—C12	1.388 (2)	C27—H27B	0.9800
C13—C14	1.501 (2)	C27—H27C	0.9800
C13—C15	1.484 (2)	C29—H29	0.9500
C15—C16	1.395 (2)	C30—H30	0.9500
C15—C20	1.398 (2)	C31—H31	0.9500
C16—C17	1.385 (2)	C32—H32	0.9500
C17—C18	1.393 (3)	C33—H33	0.9500
C18—C21	1.511 (3)	C35—H35A	0.9800
C18—C19	1.381 (3)	C35—H35B	0.9800
C19—C20	1.387 (3)	C35—H35C	0.9800
C4—H4	0.9500	C37—H37	0.9500
C6—H6B	0.9800	C38—H38	0.9500
C6—H6C	0.9800	C40—H40	0.9500
C6—H6A	0.9800	C41—H41	0.9500
C8—H8	0.9500	C42—H42A	0.9800
C9—H9	0.9500	C42—H42B	0.9800
C10—H10	0.9500	C42—H42C	0.9800
			
C1—S1—C3	90.54 (7)	C19—C20—H20	120.00
C22—S2—C24	90.44 (7)	C18—C21—H21A	109.00
C5—O3—C6	115.16 (12)	C18—C21—H21B	109.00
C26—O6—C27	116.03 (12)	C18—C21—H21C	109.00
C2—N1—C7	122.17 (13)	H21A—C21—H21B	109.00
C1—N1—C7	122.00 (12)	H21A—C21—H21C	110.00
C1—N1—C2	115.45 (13)	H21B—C21—H21C	109.00
N3—N2—C1	109.05 (12)	S2—C22—N4	112.14 (10)
N2—N3—C13	114.60 (13)	S2—C22—N5	127.04 (11)
C22—N4—C23	115.76 (11)	N4—C22—N5	120.82 (13)
C23—N4—C28	122.18 (11)	O4—C23—N4	124.89 (12)
C22—N4—C28	122.07 (12)	O4—C23—C24	125.29 (13)
N6—N5—C22	111.28 (12)	N4—C23—C24	109.82 (12)
N5—N6—C34	113.98 (13)	S2—C24—C23	111.77 (10)
S1—C1—N2	125.36 (12)	S2—C24—C25	127.74 (11)
S1—C1—N1	112.54 (11)	C23—C24—C25	120.49 (13)
N1—C1—N2	122.11 (14)	C24—C25—C26	121.26 (13)
O1—C2—N1	124.49 (14)	O5—C26—O6	125.04 (14)
O1—C2—C3	125.59 (14)	O5—C26—C25	123.98 (14)
N1—C2—C3	109.92 (13)	O6—C26—C25	110.97 (13)
S1—C3—C2	111.56 (10)	N4—C28—C29	119.09 (13)
S1—C3—C4	126.95 (11)	N4—C28—C33	119.18 (13)
C2—C3—C4	121.46 (13)	C29—C28—C33	121.73 (13)
C3—C4—C5	120.84 (13)	C28—C29—C30	118.84 (15)
O2—C5—O3	124.16 (14)	C29—C30—C31	119.92 (16)
O2—C5—C4	123.97 (14)	C30—C31—C32	120.57 (16)
O3—C5—C4	111.86 (13)	C31—C32—C33	120.02 (17)
N1—C7—C12	119.78 (13)	C28—C33—C32	118.91 (15)
C8—C7—C12	121.39 (14)	N6—C34—C35	124.61 (14)
N1—C7—C8	118.83 (13)	N6—C34—C36	116.04 (13)
C7—C8—C9	118.82 (15)	C35—C34—C36	119.35 (13)
C8—C9—C10	120.57 (16)	C34—C36—C37	121.08 (14)
C9—C10—C11	119.96 (15)	C34—C36—C41	120.86 (14)
C10—C11—C12	120.32 (16)	C37—C36—C41	118.02 (14)
C7—C12—C11	118.94 (15)	C36—C37—C38	120.91 (15)
N3—C13—C14	124.30 (14)	C37—C38—C39	121.15 (16)
N3—C13—C15	116.33 (13)	C38—C39—C40	117.78 (16)
C14—C13—C15	119.36 (13)	C38—C39—C42	121.62 (16)
C13—C15—C16	121.22 (14)	C40—C39—C42	120.60 (17)
C13—C15—C20	121.17 (15)	C39—C40—C41	121.64 (17)
C16—C15—C20	117.61 (16)	C36—C41—C40	120.49 (16)
C15—C16—C17	120.94 (16)	C24—C25—H25	119.00
C16—C17—C18	121.22 (17)	C26—C25—H25	119.00
C19—C18—C21	121.55 (17)	O6—C27—H27A	109.00
C17—C18—C19	117.95 (17)	O6—C27—H27B	110.00
C17—C18—C21	120.48 (18)	O6—C27—H27C	109.00
C18—C19—C20	121.31 (17)	H27A—C27—H27B	109.00
C15—C20—C19	120.95 (17)	H27A—C27—H27C	109.00
C3—C4—H4	120.00	H27B—C27—H27C	109.00
C5—C4—H4	120.00	C28—C29—H29	121.00
O3—C6—H6A	109.00	C30—C29—H29	121.00
O3—C6—H6B	109.00	C29—C30—H30	120.00
O3—C6—H6C	109.00	C31—C30—H30	120.00
H6A—C6—H6B	110.00	C30—C31—H31	120.00
H6A—C6—H6C	109.00	C32—C31—H31	120.00
H6B—C6—H6C	109.00	C31—C32—H32	120.00
C7—C8—H8	121.00	C33—C32—H32	120.00
C9—C8—H8	121.00	C28—C33—H33	121.00
C10—C9—H9	120.00	C32—C33—H33	121.00
C8—C9—H9	120.00	C34—C35—H35A	109.00
C9—C10—H10	120.00	C34—C35—H35B	109.00
C11—C10—H10	120.00	C34—C35—H35C	109.00
C12—C11—H11	120.00	H35A—C35—H35B	109.00
C10—C11—H11	120.00	H35A—C35—H35C	109.00
C7—C12—H12	121.00	H35B—C35—H35C	110.00
C11—C12—H12	121.00	C36—C37—H37	120.00
C13—C14—H14A	109.00	C38—C37—H37	119.00
C13—C14—H14B	110.00	C37—C38—H38	119.00
C13—C14—H14C	109.00	C39—C38—H38	119.00
H14A—C14—H14B	109.00	C39—C40—H40	119.00
H14A—C14—H14C	109.00	C41—C40—H40	119.00
H14B—C14—H14C	109.00	C36—C41—H41	120.00
C17—C16—H16	120.00	C40—C41—H41	120.00
C15—C16—H16	119.00	C39—C42—H42A	109.00
C16—C17—H17	119.00	C39—C42—H42B	109.00
C18—C17—H17	119.00	C39—C42—H42C	109.00
C20—C19—H19	119.00	H42A—C42—H42B	110.00
C18—C19—H19	119.00	H42A—C42—H42C	109.00
C15—C20—H20	120.00	H42B—C42—H42C	109.00
			
C3—S1—C1—N1	0.11 (11)	C3—C4—C5—O3	178.91 (14)
C3—S1—C1—N2	−179.71 (14)	N1—C7—C8—C9	−179.64 (15)
C1—S1—C3—C2	−0.07 (11)	C12—C7—C8—C9	0.5 (2)
C1—S1—C3—C4	177.97 (15)	N1—C7—C12—C11	179.59 (14)
C24—S2—C22—N4	−1.44 (11)	C8—C7—C12—C11	−0.5 (2)
C24—S2—C22—N5	178.62 (15)	C7—C8—C9—C10	−0.2 (3)
C22—S2—C24—C23	−0.07 (10)	C8—C9—C10—C11	−0.1 (3)
C22—S2—C24—C25	179.95 (14)	C9—C10—C11—C12	0.0 (3)
C6—O3—C5—C4	176.87 (12)	C10—C11—C12—C7	0.3 (3)
C6—O3—C5—O2	−2.7 (2)	N3—C13—C15—C16	5.6 (2)
C27—O6—C26—C25	177.82 (12)	C14—C13—C15—C20	5.1 (2)
C27—O6—C26—O5	−1.2 (2)	N3—C13—C15—C20	−175.20 (15)
C2—N1—C1—S1	−0.13 (16)	C14—C13—C15—C16	−174.20 (15)
C7—N1—C2—C3	173.02 (13)	C13—C15—C20—C19	−177.58 (15)
C1—N1—C7—C8	90.13 (18)	C13—C15—C16—C17	177.81 (15)
C1—N1—C7—C12	−89.96 (18)	C16—C15—C20—C19	1.7 (2)
C2—N1—C7—C8	−82.35 (19)	C20—C15—C16—C17	−1.5 (2)
C2—N1—C7—C12	97.56 (18)	C15—C16—C17—C18	0.1 (3)
C1—N1—C2—O1	−179.89 (15)	C16—C17—C18—C19	1.0 (3)
C1—N1—C2—C3	0.08 (17)	C16—C17—C18—C21	−177.52 (18)
C7—N1—C2—O1	−7.0 (2)	C21—C18—C19—C20	177.73 (18)
C7—N1—C1—N2	6.8 (2)	C17—C18—C19—C20	−0.8 (3)
C2—N1—C1—N2	179.70 (14)	C18—C19—C20—C15	−0.6 (3)
C7—N1—C1—S1	−173.08 (11)	O4—C23—C24—S2	−178.30 (12)
N3—N2—C1—N1	−178.21 (13)	O4—C23—C24—C25	1.7 (2)
C1—N2—N3—C13	−178.90 (13)	N4—C23—C24—S2	1.55 (15)
N3—N2—C1—S1	1.60 (18)	N4—C23—C24—C25	−178.47 (14)
N2—N3—C13—C14	0.8 (2)	S2—C24—C25—C26	−2.0 (2)
N2—N3—C13—C15	−178.93 (12)	C23—C24—C25—C26	178.07 (13)
C23—N4—C22—N5	−177.30 (14)	C24—C25—C26—O5	−1.4 (2)
C28—N4—C22—S2	−176.83 (11)	C24—C25—C26—O6	179.53 (14)
C23—N4—C22—S2	2.75 (16)	N4—C28—C29—C30	179.08 (14)
C22—N4—C23—O4	177.12 (14)	C33—C28—C29—C30	−0.1 (2)
C22—N4—C23—C24	−2.73 (17)	N4—C28—C33—C32	−178.52 (14)
C28—N4—C23—O4	−3.3 (2)	C29—C28—C33—C32	0.6 (2)
C28—N4—C23—C24	176.84 (12)	C28—C29—C30—C31	−0.1 (2)
C22—N4—C28—C29	110.72 (17)	C29—C30—C31—C32	−0.3 (3)
C22—N4—C28—C33	−70.12 (19)	C30—C31—C32—C33	0.9 (3)
C28—N4—C22—N5	3.1 (2)	C31—C32—C33—C28	−1.0 (3)
C23—N4—C28—C33	110.33 (17)	N6—C34—C36—C37	−6.3 (2)
C23—N4—C28—C29	−68.83 (19)	N6—C34—C36—C41	171.27 (15)
N6—N5—C22—S2	3.38 (19)	C35—C34—C36—C37	174.39 (15)
N6—N5—C22—N4	−176.57 (13)	C35—C34—C36—C41	−8.0 (2)
C22—N5—N6—C34	−165.53 (14)	C34—C36—C37—C38	177.40 (15)
N5—N6—C34—C36	−175.22 (13)	C41—C36—C37—C38	−0.3 (2)
N5—N6—C34—C35	4.0 (2)	C34—C36—C41—C40	−176.81 (15)
O1—C2—C3—C4	1.8 (2)	C37—C36—C41—C40	0.9 (2)
O1—C2—C3—S1	179.97 (14)	C36—C37—C38—C39	−0.7 (3)
N1—C2—C3—S1	0.02 (16)	C37—C38—C39—C40	1.1 (3)
N1—C2—C3—C4	−178.15 (14)	C37—C38—C39—C42	−179.48 (18)
C2—C3—C4—C5	177.02 (14)	C38—C39—C40—C41	−0.5 (3)
S1—C3—C4—C5	−0.8 (2)	C42—C39—C40—C41	−179.92 (18)
C3—C4—C5—O2	−1.5 (2)	C39—C40—C41—C36	−0.5 (3)

### Hydrogen-bond geometry (Å, º)

Cg is the centroid of the C36–C41 ring.

**Table:**

*D*—H···*A*	*D*—H	H···*A*	*D*···*A*	*D*—H···*A*
C4—H4···O6^i^	0.95	2.56	3.4802 (19)	163
C6—H6*B*···O4^i^	0.98	2.52	3.465 (2)	163
C8—H8···O2^ii^	0.95	2.56	3.359 (2)	142
C12—H12···O4	0.95	2.43	3.302 (2)	152
C14—H14*A*···N2	0.98	2.28	2.728 (2)	107
C27—H27*B*···O1^i^	0.98	2.45	3.410 (2)	166
C30—H30···O5^iii^	0.95	2.44	3.331 (2)	157
C32—H32···O1^iv^	0.95	2.57	3.340 (2)	139
C35—H35*A*···N5	0.98	2.27	2.718 (2)	106
C33—H33···*Cg*^v^	0.95	2.58	3.4951 (19)	162

Symmetry codes: (i) −*x*+2, −*y*, −*z*+1; (ii) −*x*+1, −*y*, −*z*+1; (iii) −*x*+1, *y*+1/2, −*z*+1/2; (iv) *x*, −*y*+1/2, *z*−1/2; (v) −*x*+1, −*y*, −*z*.

## References

[bb1] Allen, F. H., Kennard, O., Watson, D. G., Brammer, L., Orpen, A. G. & Taylor, R. (1987). *J. Chem. Soc. Perkin Trans. 2*, pp. S1–19.

[bb2] Babaoğlu, K., Page, M. A., Jones, V. C., McNeil, M. R., Dong, C., Naismith, J. H. & Lee, R. E. (2003). *Bioorg. Med. Chem. Lett.* **13**, 3227–3230. 10.1016/s0960-894x(03)00673-5 12951098

[bb3] Barreca, M. L., Balzarini, J., Chimirri, A., Clercq, E. D., Luca, L. D., Höltje, H. D., Höltje, M., Monforte, A. M., Monforte, P., Pannecouque, C., Rao, A. & Zappala, M. (2002). *J. Med. Chem.* **45**, 5410–5413. 10.1021/jm020977+ 12431069

[bb4] Botti, P., Pallin, T. D. & Tam, J. P. (1996). *J. Am. Chem. Soc.* **118**, 10018–10024.

[bb5] Brandenburg, K. & Putz, H. (2012). *DIAMOND* Crystal Impact GbR, Bonn, Germany.

[bb6] Bruker (2013). *APEX2*, *SAINT* and *SADABS* Bruker AXS Inc., Madison, Wisconsin, USA.

[bb7] Çapan, G., Ulusoy, N., Ergenç, N. & Kiraz, M. (1999). *Monatsh. Chem.* **130**, 1399–1407.

[bb8] Farrugia, L. J. (2012). *J. Appl. Cryst.* **45**, 849–854.

[bb9] Pandey, Y., Sharma, P. K., Kumar, N. & Singh, A. (2011). *Int. J. Pharm. Tech. Res.* **3**, 980–985.

[bb10] Pfahl, M., Al-Shamma, H. A., Fanjul, A. N., Pleynet, D. P. M., Bao, H., Spruce, L. W., Cow, C. N., Tachdjian, C., Zapt, J. W. & &Wiemann, T. R. (2003). Patent WO 2003/050 098; Int. Appl. No. PCT/US2002/039 178.

[bb11] Sayyed, M., Mokle, S., Bokhare, M., Mankar, A., Surwase, S., Bhusare, S. & Vilohute, Y. (2006). *Arkivoc*, **ii**, 187–197.

[bb12] Sharma, R., Nagda, D. P. & Talesara, G. L. (2006). *Arkivoc*, **i**, 1–12.

[bb13] Sheldrick, G. M. (2008). *Acta Cryst.* A**64**, 112–122. 10.1107/S0108767307043930 18156677

[bb14] Spek, A. L. (2009). *Acta Cryst.* D**65**, 148–155. 10.1107/S090744490804362X PMC2631630 19171970

